# The association between vaccine coverage and herd protection: exploratory analyses of a cluster-randomised trial of Vi conjugate vaccine

**DOI:** 10.1016/j.eclinm.2026.103863

**Published:** 2026-04-15

**Authors:** Yiyuan Zhang, Faisal Ahmmed, Farhana Khanam, Prasanta Kumar Biswas, Yama F. Mujadidi, Sarah Kelly, Firdausi Qadri, John D. Clemens, Andrew J. Pollard, Xinxue Liu

**Affiliations:** a Oxford Vaccine Group, Department of Paediatrics, University of Oxford, Oxford, UK; b NIHR Oxford Biomedical Research Centre and Oxford University Hospitals NHS Foundation Trust, Oxford, UK; c International Centre for Diarrhoeal Disease Research, Bangladesh (icddr,b), Dhaka, Bangladesh; d International Vaccine Institute, Seoul, South Korea; e UCLA Fielding School of Public Health, Los Angeles, CA, 90095-1772, USA

**Keywords:** Typhoid conjugate vaccine, Herd protection, Vaccine coverage, Indirect vaccine effectiveness, Cluster-randomised trial, Bangladesh

## Abstract

**Background:**

Indirect protection from the typhoid conjugate vaccine (TCV) has been negligible, possibly due to vaccination being restricted to children, which may have limited the overall vaccine coverage needed to sufficiently interrupt transmission dynamics across endemic populations. In this study herd protection was evaluated in households and schools with different TCV coverages by conducting secondary analyses of a double-blind cluster-randomised trial on TCV in Bangladesh.

**Methods:**

TyVAC Bangladesh was a cluster-randomised trial conducted in Dhaka, Bangladesh between April 2018 and March 2020 (ISRCTN11643110 at www.isrctn.com). For this study, 150 geographical clusters were randomly assigned to receive TCV or Japanese encephalitis (JE) vaccine. Children aged 9 months to <16 years were vaccinated. Household-level herd protection was estimated by indirect vaccine effectiveness (iVE), comparing typhoid incidence among residents of all ages who did not receive the assigned study vaccine in TCV clusters with those in JE clusters using mixed-effects Poisson regression. The iVE was calculated as (1 − adjusted incidence rate ratio [aIRR]) × 100%. Household-level coverage was stratified by the number of vaccinated children (0, 1, ≥2) and the presence of eligible children. School-level herd protection was evaluated among students attending 35 primary and secondary schools who did not receive TCV by comparing typhoid incidence in high-coverage (>median) versus low-coverage schools (≤median), where school-level coverage was measured by the proportion of TCV-vaccinated students. The aIRR was estimated by mixed-effects Poisson regression.

**Findings:**

Household-level analysis included 129,601 unvaccinated residents from 40,435 households in the TCV arm and 129,973 unvaccinated residents from 40,577 households in the JE arm (median household size: 4.0 [IQR: 3.0, 5.0]). During 24-month follow-up between April 2018 and March 2020, the overall aIRR comparing all unvaccinated residents in TCV versus JE clusters was 0.91 [95% CI: 0.43, 1.91]. Stratified by household vaccine coverage, aIRR was 0.81 [95% CI: 0.15, 4.52] in households without eligible children and 0.97 [0.31, 3.04] in households with ≥ 1 eligible children but none vaccinated, and iVE was 25% [95% CI: −234, 83] and −26% [−766, 82] in households with ≥ 1 eligible children and 1, or ≥ 2 vaccinated children, respectively, with no significant interaction between number of vaccinees and arm (p = 0.85). School-level analysis (9209 children, 8%–55% TCV coverage) showed typhoid incidence of 349 [95% CI: 219, 528] and 329 [180, 552] per 100,000 person-years among non-TCV vaccinated students in low- and high-coverage schools, respectively (aIRR: 0.96 [95% CI: 0.28, 3.37]).

**Interpretation:**

Our study did not find significant herd protection across vaccine coverage levels in households and schools, although estimates were imprecise with wide confidence intervals.

**Funding:**

Gates Foundation.

**Research in context**

**Evidence before this study**

We conducted a PubMed search on May 8, 2025, using the terms “vaccine coverage” AND “typhoid” AND (“herd protection” OR “indirect protection”), which identified two publications. A cluster-randomised trial in India demonstrated that a single 0.5 ml dose of Vi polysaccharide vaccine containing 25 ug Vi Polysaccharide (Typherix, GlaxoSmithKline) with 61% coverage in persons aged ≥ 2 conferred 44% protection to unvaccinated residents, providing evidence that indirect protection can occur. However, we did not identify studies that specifically investigated the association between varying levels of typhoid vaccine coverage and the magnitude of herd protection effects.

**Added value of this study**

To our best knowledge, this study provides the first investigation of the relationship between typhoid vaccine coverage and herd protection across varying coverage levels. Using data from a cluster-randomised trial of Vi conjugate vaccine, we analysed whether increasing vaccine coverage in households and schools corresponded with greater indirect protection of unvaccinated individuals and found no significant herd protection effects at any of the coverage levels examined.

**Implications of all the available evidence**

The herd protection effects of typhoid vaccination may be context-dependent, potentially influenced by local transmission patterns, environmental factors, or population density that differ from settings where indirect effects have previously been observed. These findings highlight the importance for policymakers and public health officials to consider this heterogeneity and develop appropriate implementation strategies for typhoid vaccination programs. In regions with epidemiological characteristics similar to our study setting, higher coverage may be required to effectively interrupt overall circulation. Vaccination campaign targeting children is likely to be insufficient to block ongoing transmission. Expanding vaccination to wider population segments or targeting key sources of transmission could therefore be considered to achieve meaningful population-level control.

## Introduction

Typhoid fever, a systemic infection caused by *Salmonella* enterica serovar Typhi (*Salmonella* Typhi), continues to impose a significant burden on low- and middle-income countries. Characterised by fever, abdominal pain, and various systemic symptoms, typhoid fever is primarily transmitted through the faecal-oral route. The disease disproportionately affects populations in areas with poor sanitation and limited access to clean water, making it a key indicator of socioeconomic disparities in public health. In 2021, 9.32 million cases of enteric fever were reported, resulting in 107,000 deaths.^1^ This highlights its substantial global health burden and the need for continued prevention and control efforts. The growing threat of antimicrobial-resistant *Salmonella* Typhi strains, particularly the extensively drug-resistant (XDR) strain, first identified in Pakistan in 2016, has complicated treatment options and heightened concerns about disease control. In addition, azithromycin-resistant *S*. Typhi strains have emerged in Bangladesh, further limiting effective oral treatment options and underscoring the urgency of strengthened prevention strategies.^2^ In response, the implementation of effective typhoid vaccines has become a global health priority. So far, there are three typhoid conjugate vaccines (TCVs) pre-qualified by the World Health Organisation (WHO), and ten countries have introduced TCV till 2025.^3^

While direct protection by TCV has been well-established, the indirect (herd) protection remains unclear, which is a critical factor for population-level control. To address this, studies have employed innovative analytical approaches, such as the “fried egg” approach, to assess potential herd protection.^4^, ^5^, ^6^ This technique examines progressively smaller inner areas of clusters (the “yolks”) to minimise the influence of inward disease transmission from outside the clusters. In a cluster-randomised trial (CRCT) of TCV in Bangladesh (TyVAC Bangladesh), this approach failed to reveal significant herd protection, as vaccine effectiveness estimates neither increased when restricted to central subclusters nor reached statistical significance when considering the entire population.^7^ Conversely, in a CRCT of a Vi polysaccharide vaccine in Kolkata, this approach demonstrated higher total and overall protection in the innermost subclusters and significant indirect protection for the entire cluster but failed to show a clear trend for indirect protection due to limited sample size.^8^ These findings highlight the challenges of detecting herd protection, or its limited presence in densely populated urban settings where transmission dynamics are complex, although it has been observed, as demonstrated by the study in Kolkata. This suggests that while detection is challenging, the presence and magnitude of herd protection can be highly variable and dependent on factors such as vaccine type and local transmission dynamics.

One possible explanation for the difference in herd protection between the two trials is vaccine coverage. In Kolkata, residents aged 2 years and older were offered the study vaccine, while the eligible population for vaccination in Bangladesh was 9 months–15 years, leading to approximately 57% and 20% vaccine coverage among all residents, respectively.^6^^,^^8^ While typhoid is mainly transmitted by contaminated food and water sources, the possibility of person-to-person transmission cannot be ruled out. Saul et al. (2013) developed mathematical models to explore the impact of person-to-person transmission on vaccine effectiveness, suggesting that high coverage rates may be necessary to achieve significant population-level protection in areas with substantial carrier populations because carriers represent an ongoing source of infection that persists even when person-to-person transmission is reduced through partial vaccination.^9^ However, some evidence suggests that in very high transmission settings, ongoing transmission may be driven more by individuals with acute or recent infections than by chronic carriers.^10^

This study aims to further evaluate the impact of vaccine coverage on herd protection at the household and school level by conducting a secondary analysis using TyVAC Bangladesh trial data, providing critical information for future cost-effective vaccination strategies and policy decisions in typhoid-endemic regions.

## Methods

### TyVAC Bangladesh trial

TyVAC Bangladesh (ISRCTN11643110) was a participant-blind and observer-blind cluster randomised trial in Dhaka, Bangladesh which has been previously reported.^6^ The study area was divided into 150 contiguous geographic clusters with similar population sizes based on a baseline census of the entire population in the study area between 14th February and 25th March 2018. These clusters were then randomised (1:1) to either a single 0.5 ml dose of TCV containing 25 ug Vi Polysaccharide (Typbar TCV, Bharat Biotech International, Hyderabad, India) vaccine or a single 0.5 mL dose of JE (SA-14-14-2 Japanese encephalitis, Chengdu Institute of Biological Products, China) vaccine as the control. Full details of the randomisation and blinding, including sequence generation and allocation concealment, are provided in the primary trial publication.^6^ Eligible children, aged 9 months to <16 years, were offered a single dose of the vaccine randomised to their cluster of residence. The baseline vaccination was done between 15th April and 15th May 2018. At six-month intervals during the two years after baseline, census updates of the population were conducted in all clusters, ensuring up-to-date household information. Children who fulfilled the eligibility criteria for vaccination and had not received the study vaccines allocated to the cluster were offered the vaccine in the catch-up campaign after each census update. After completing the trial, school attendance data was retrospectively collected during a census update. We began by reviewing all 332 schools in the study area. From this initial review, we purposively selected 35 formal primary and secondary schools that were active and had over 100 students, given the large number of schools in the area. Children were then asked to report their attendance at these pre-selected schools or other institutions. This school attendance information was subsequently linked to each child's vaccination status using their unique identification number.

The surveillance system for enteric fever was established in February 2018, prior to the vaccination campaign. Participants in the study area presented at any of the eight designated clinical facilities with at least two days of fever or with an axillary temperature of at least 38 °C were recruited into the trial after providing informed consent, regardless of their vaccination status. A blood specimen was collected for each fever presentation, and blood culture results were reported as negative or positive for *Salmonella* Typhi, *Salmonella* Paratyphi, non-typhoidal *Salmonella*, and other pathogens. The detailed methodology for blood specimen culture has been thoroughly described in the previous publication.^6^ The surveillance system was suspended on 15th March 2020 due to the COVID-19 pandemic, and the data collected between baseline vaccination and 15th March 2020 were used in this study.

The TyVAC trial received ethical approval from the Research and Ethical Review Committees of the International Centre for Diarrhoeal Disease Research, Bangladesh (icddr,b), as well as the institutional review boards of Oxford University, Oxford, UK. This study was a pre-specified secondary objective covered by the original TyVAC trial's ethical approval. Written informed consent was obtained from all participants, including parent or guardian consent for all those younger than 16 years and participant assent for those aged 11 to less than 16 years.

### Outcomes

The primary outcome of this study was the occurrence of blood-culture-confirmed typhoid fever, defined as a febrile episode with at least one blood culture positive *Salmonella* Typhi. Febrile episodes were counted if fever onset occurred one or more days after follow-up started (defined in the statistical analysis section). Episodes were grouped as a single episode if fever onset dates fell within 14 days of discharge from the prior episode. We used blood culture-confirmed infection by pathogens other than *Salmonella* Typhi as a bias indicator, which was defined as a febrile episode in which at least one blood culture was positive for *Salmonella* Paratyphi, non-typhoidal Salmonella, or other pathogens. Only the first episode of either typhoid fever or an infection by another pathogen was included.

### Study design

The impact of vaccine coverage on herd protection was investigated at two settings, namely the household/family-level and school-level.

In the family-level analysis, families in the study area were categorised into four groups based on the number of age-eligible children for vaccination (between 9 months and <16 years at any of the vaccination campaigns) and the number of vaccinated children (either TCV-recipients or JE-recipients). The four groups are families with no age-eligible children (family group 1), families with at least one age-eligible child but none vaccinated (family group 2), families with at least one age-eligible child and one child vaccinated (family group 3), and families with more than one age-eligible child and two or more children vaccinated (family group 4). Family groups 1 and 2, which had no vaccinated children, served as baseline comparators between the TCV and JE arms. We separated them to account for potential differences in health-seeking behaviour based on the presence of an age-eligible child. We hypothesised that as the number of TCV-vaccinated children in a household increases, the herd protection for unvaccinated household members (by comparing the incidence of typhoid fever between TCV and JE groups) also increases.

The school-level analysis focused on children who attended the pre-selected 35 schools. These schools spanned both TCV and JE clusters, so vaccine coverage at school was defined as the percentage of post-vaccination follow-up time of TCV-vaccinated children over the follow-up period for all children. The follow-up period for all children started at the date of residence and ended at the earliest occurrence of death, 15th March 2020, or the date of moving out of the study area. The date of residence was defined as the midpoint of the baseline vaccination campaign (30th April, 2018) for unvaccinated children living in the study area at that time, the date of vaccination for children vaccinated at baseline, or the date of first migration into or birth in the study area for in-migrants and births post-baseline. The 35 schools were dichotomised into high-coverage (>median) and low-coverage (≤median) groups by the median school TCV coverage rate. Our hypothesis is that the incidence of typhoid fever in TCV-unvaccinated children is lower in schools with high TCV coverage compared to those with low TCV coverage.

### Statistical analysis

No formal sample size or statistical power calculation was performed for this study, as the sample size was determined by the original cluster-randomised trial design.

The population for the analyses at the family level was non-vaccinees in the four family groups defined as residents who were not vaccinated during any of the vaccination campaigns. Subgroup analyses in non-vaccinees were also conducted by age at the date of residence (>18 years vs. ≤18 years). This date was defined as the midpoint of the baseline vaccination campaign (30th April, 2018) for residents living in the study area at baseline or the date of first migration into or birth in the study area for in-migrants and births post-baseline. The follow-up period of non-vaccinees in family groups 1 and 2 began from their date of residence. The follow-up period of non-vaccinees in family groups 3 and 4 started from the earliest vaccination date of children in the same family. The follow-up period of non-vaccinees in all family groups ended on the date of death, 15th March 2020, or the date of moving out of the study arm, whichever occurred first.

The adjusted incidence rate ratio of typhoid (aIRR) among non-vaccinees in the TCV arm compared to the JE arm within every family group was estimated using a mixed-effects Poisson regression model as the origin study was cluster-randomised. The model was adjusted for the stratification factors of the original cluster randomisation (the number of children aged 9 months to <16 years in the cluster, ward, and distance of cluster to the nearest health facility), demographics (age, sex, toilet type in the house, drinking water source, treatment of drinking water, and handwashing practices before meals and after defecation), follow-up period and family size as fixed effects, with random intercepts for clusters and families. Missing demographics data were minimal (<1%), and no imputation was performed. The iVE was calculated as (1-aIRR) ∗ 100. To evaluate the difference in iVE between families with different numbers of vaccinated children, the interaction between the number of vaccinees in a household and the vaccine arm was evaluated. Two mixed-effects Poisson regression models were fitted for non-vaccinees in all family groups (both adjusted for the above variables and the number of vaccinees; one with and one without this interaction term), and a likelihood ratio test between these two models was then conducted to determine interaction.

For sensitivity analysis, we included the follow-up period before vaccination and considered family groups 3 and 4 during follow-up period between the date of residence and the earliest vaccination date of children in the same family as family group 2. Additionally, we estimated the aIRR using a simplified mixed-effects Poisson regression model, which adjusted for three stratification factors, follow-up period, age, sex, family size as fixed effects, with random intercepts for clusters and families. Finally, we revised the criteria for categorising families, shifting from the number of vaccinees to the proportion of vaccinees among age-eligible children within a family. Family group 1 continued to include families with no age-eligible children, consistent with our main analysis. Group 2 comprised families with a 0% proportion of vaccinees, meaning they had at least one age-eligible child but none were vaccinated—a definition also aligned with our main analysis. Group 3 encompassed families with a proportion of vaccinees between 1% and 50%, while group 4 included families with a proportion of vaccinees between 51% and 100%. Potential biases were examined by calculating the aIRR of blood culture-confirmed infection by pathogens other than *Salmonella* Typhi in non-vaccinees by the same mixed-effects Poisson regression. We also evaluated the total vaccine protection of TCV among vaccinees in family groups 3 and 4 using the same model. Vaccinees were defined as residents who received either TCV or JE during any of the vaccination campaigns. The follow-up period of vaccinees started on the date of vaccination and ended on the date of death, 15th March 2020, or the date of moving out of the study arm, whichever occurred first.

The primary population for the analysis at the school level was non-TCV recipients. Because the 35 pre-selected schools enrolled children from both TCV and JE arms, school-level vaccine coverage was evaluated by including all children, including TCV vaccinees, JE vaccinees and non-vaccinees. As the JE vaccine does not provide protection against typhoid, JE recipients were grouped with non-vaccinees as non-TCV recipients for this analysis. Therefore, non-TCV recipients included all children in JE clusters (regardless of JE vaccination status) and children in TCV clusters who either did not receive TCV or received TCV during catch-up campaigns. For children in JE clusters and those in TCV clusters who did receive TCV, the follow-up time started at the midpoint of the baseline vaccination campaign (April 30, 2018) for unvaccinated children living in the study area at that time, the date of vaccination for children vaccinated at baseline, or the date of first migration into or birth in the study area for in-migrants and births post-baseline, and ended at the earliest occurrence of death, 15th March 2020, or the date of moving out of the study area. For children in TCV clusters who received TCV during catch-up campaigns, we only included the pre-vaccination follow-up period in the analysis as non-TCV vaccinees. The aIRR of typhoid among non-TCV recipients in the high-coverage schools compared to those in the low-coverage schools was estimated using a mixed-effects Poisson regression model adjusted for the same set of stratification factors and demographics as in the family-level analysis, follow-up period, and the arm (JE or TCV) where schools were located in with random intercepts for clusters and schools. For sensitivity analysis, we estimated the aIRR using a simplified mixed-effects Poisson regression model, which adjusted for stratification factors, follow-up period, age, sex, and arm of schools with random intercepts for clusters and schools.

In addition to the primary analysis of non-TCV recipients, we conducted similar analyses to the family-level analysis to assess potential bias and total vaccine protection. These included estimating the aIRR of blood culture-confirmed infection by pathogens other than *Salmonella* Typhi in non-TCV recipients (using the same follow-up period as for typhoid cases) and the aIRR of blood culture-confirmed typhoid in TCV recipients. TCV recipients were children receiving TCV during any of the vaccination campaigns, and the follow-up period used to calculate the aIRR of blood culture-confirmed typhoid was their post-vaccination follow-up time. Notably, children receiving TCV during catch-up campaigns were included in both the analysis of non-TCV recipients and of TCV recipients. Their febrile episodes before and after vaccination were analysed as non-TCV recipients and TCV recipients, respectively.

All analyses were performed in R 4.2.2.

### Role of the funding source

This project was funded by the Gates Foundation. The funder of the study had no role in study design, data collection, data analysis, data interpretation, or writing of the paper.

## Results

In the baseline and catch-up vaccination campaigns, 33,822 children received TCV vaccine and 33,398 children received JE vaccine. There were 81,360 families in the study area captured during two years of follow-up, of which 348 were excluded from the household-level analysis due to having no unvaccinated residents. This resulted in 40,435 families in the TCV arm and 40,577 families in the JE arm (Table 1). Families had a median size of 4.0 [IQR: 3.0, 5.0] in both arms and were balanced in terms of the household hygiene factors (Table 1). The unvaccinated population in the four family groups comprised 129,601 individuals in the TCV arm (105,374 adults and 24,227 children) and 129,973 individuals in the JE arm (105,837 adults and 24,136 children). The unvaccinated adults and children were well-balanced between the two study arms (Table 1).

**Table 1:** Demographic characteristics of the unvaccinated residents.

Household/Family-level	JE	TCV
	N = 40,577	N = 40,435
Family size (median IQR)	4.0 (3.0–5.0) [n = 40,577]	4.0 (3.0–5.0) [n = 40,435]
Family group		
Group 1	10,732	10,466
Family size (median range)	2 (1–13)	2 (1–12)
Number of age-eligible children (median range)	0 (0–0)	0 (0–0)
Number of vaccinated children (median range)	0 (0–0)	0 (0–0)
Family vaccine coverage^a^ (median range)	0 (0–0)	0 (0–0)
Group 2	8970	8861
Family size (median range)	4 (1–23)	4 (1–19)
Number of age-eligible children (median range)	1 (1–6)	1 (1–6)
Number of vaccinated children (median range)	0 (0–0)	0 (0–0)
Family vaccine coverage^a^ (median range)	0 (0–0)	0 (0–0)
Group 3	11,278	11,444
Family size (median range)	4 (2–15)	4 (2–14)
Number of age-eligible children (median range)	1 (1–6)	1 (1–6)
Number of vaccinated children (median range)	1 (1–1)	1 (1–1)
Family vaccine coverage^a^ (median range)	0.25 (0.07–0.50)	0.25 (0.07–0.50)
Group 4	9597	9664
Family size (median range)	5 (3–24)	5 (3–19)
Number of age-eligible children (median range)	2 (2–10)	2 (2–11)
Number of vaccinated children (median range)	2 (2–6)	2 (2–9)
Family vaccine coverage^a^ (median range)	0.50 (0.08–0.80)	0.50 (0.12–0.83)
Ward		
2	16,169 (39.8%)	16,737 (41.4%)
3	9157 (22.6%)	10,567 (26.1%)
5	15,251 (37.6%)	13,131 (32.5%)
Distance to study clinics (metre, median IQR)	407.7 (268.3–528.3) [n = 40,577]	401.1 (252.5–564.9) [n = 40,435]
Type of toilet		
Flush toilet	2198 (5.4%)	2400 (5.9%)
Others	38,379 (94.6%)	38,035 (94.1%)
Source of drinking water		
Own water source	11,321 (27.9%)	10,464 (25.9%)
Others	29,256 (72.1%)	29,971 (74.1%)
Type of drinking water		
Treated drinking water^b^	30,623 (75.5%)	29,811 (73.7%)
Not treated	9948 (24.5%)	10,623 (26.3%)
NA	6 (0.0%)	1 (0.0%)
Wash hand with soap before meal		
Yes	30,359 (74.8%)	30,776 (76.1%)
No	10,218 (25.2%)	9659 (23.9%)
Wash hand with soap after defecation		
Yes	39,604 (97.6%)	39,459 (97.6%)
No	973 (2.4%)	976 (2.4%)
Have monthly saving		
Yes	8531 (21.0%)	8835 (21.9%)
No	31,436 (77.5%)	31,076 (76.9%)
NA	610 (1.5%)	524 (1.3%)
Monthly approximate HH expenditure (US Dollar, median, IQR)	138.2 (105.6–187.2) [n = 40,502]	140.2 (105.6–192.0) [n = 40,427]

Individual-level	Adults^c^	
	JE	TCV
	N = 105,837	N = 105,374
Age (median, IQR)	32.4 (25.2–43.0) [n = 105,837]	32.3 (25.1–43.0) [n = 105,374]
Sex		
Male	52,091 (49.2%)	51,816 (49.2%)
Female	53,746 (50.8%)	53,558 (50.8%)
Attends school		
Yes	5852 (5.5%)	5740 (5.4%)
No	99,316 (93.8%)	98,345 (93.3%)
Unknown	669 (0.6%)	1289 (1.2%)
Religion		
Muslim	104,458 (98.8%)	103,679 (98.5%)
Others	1236 (1.2%)	1555 (1.5%)

Individual-level	Children^c^	
	JE	TCV
Age (median, IQR)	N = 24,136	N = 24,227
Sex		
Male	12,311 (51.0%)	12,454 (51.4%)
Female	11,825 (49.0%)	11,773 (48.6%)
Attends school		
Yes	11,035 (45.7%)	10,973 (45.3%)
No	5561 (23.0%)	5594 (23.1%)
Unknown	7540 (31.2%)	7660 (31.6%)
Religion		
Muslim	23,885 (99.0%)	23,899 (98.6%)
Others	251 (1.0%)	328 (1.4%)

1.00 Bangladeshi Taka = 0.0092 US Dollar (the rate on 18th July 2023).

a Number of vaccinated children/family size.

b Including boiled, filtered, and chemical treated drinking water.

c Adults were defined as residents aged over 18. Children were defined as residents aged younger than or equal to 18.

Among all residents who did not receive the assigned study vaccine, the overall adjusted incidence rate ratio (aIRR) comparing TCV versus JE clusters was 0.91 [95% CI: 0.43, 1.91]. The aIRR for unvaccinated residents were 0.81 [95% CI: 0.15, 4.52] in group 1 and 0.97 [0.31, 3.04] in group 2. The iVE for unvaccinated residents was 25% [95% CI: −234, 83] in group 3 and −26% [95% CI: −766, 82] in group 4 (Table 2). The interaction between the number of vaccinees in a household and the vaccine arm was not statistically significant (p for interaction = 0.85). In the sensitivity analysis, including family groups 3 and 4 during the follow-up period between date of residence and the earliest vaccination date in the same family as family group 2, resulted in a minimal change to the aIRR in family group 2 (0.90 [95% CI: 0.33, 2.45]) (Table 2). Furthermore, a simplified model yielded aIRR and/or iVE estimates similar to the initial findings (Table 2). Importantly, the re-categorisation of families (from the number of vaccinees to the proportion of vaccinees among age-eligible children) did not alter the finding: for unvaccinated residents, no significant differences in incidence rates were observed between the TCV and JE arms in any of the newly defined groups (Table S1). Analysis of infections by pathogens other than *Salmonella* Typhi at the family level revealed no significant differences in incidence rates between unvaccinated residents in the JE and TCV arms. The aIRRs of other infections were 0.40 [95% CI: 0.14, 1.13], 0.66 [0.34, 1.30], 1.02 [0.11, 9.74], and 1.58 [0.09, 28.51] in family groups 1, 2, 3, and 4, respectively (Table 3). No significant interaction was found (p = 0.57) (Table 3). Similar results were seen when the analyses were conducted in adults and children separately for both typhoid fever and other infections (Table 2, Table 3).

**Table 2:** Incidence of blood culture-confirmed typhoid fever among non-vaccinees by family vaccine coverage.^a^

	Family group	With age-eligible children	Number of vaccinees	N/PYs (n)^b^		Incidence, per 100,000 PYs		Adjusted incidence rate ratio (%) [95% CI]^d^	Vaccine effectiveness (%) [95% CI]^d^	P value	P value for interaction^e^	Adjusted incidence rate ratio (%) [95% CI] (simplified)^g^
				JE	TCV	JE	TCV					
All family members	1	No	0	27/33,725 (n = 29,276)	21/32,431 (n = 28,451)	80	65	0.81 [0.15, 4.52]		0.81	0.85	0.81 [0.15, 4.56]
	2	Yes	0	52/42,807 (n = 37,485)	47/41,259 (n = 36,700)	121	114	0.97 [0.31, 3.04]		0.96		1.02 [0.32, 3.22]
	2^f^	Yes	0	73/52,510 (n = 74,410)	62/50,948 (n = 74,546)	139	122	0.90 [0.33, 2.45]		0.84		0.91 [0.33, 2.49]
	3	Yes	1	34/41,723 (n = 34,695)	26/42,923 (n = 35,347)	81	61	0.75 [0.17, 3.34]	25 [−234, 83]	0.71		0.76 [0.17, 3.38]
	4	Yes	2 and more	16/38,728 (n = 28,517)	21/38,899 (n = 29,103)	41	54	1.26 [0.18, 8.66]	−26 [−766, 82]	0.81		1.26 [0.19, 8.60]
	1 & 2		0	79/76,531 (n = 66,761)	68/73,689 (n = 65,151)	103	92	0.90 [0.35, 2.35]		0.84		0.92 [0.35, 2.39]
	3 & 4	Yes	1 and more	50/80,451 (n = 63,212)	47/81,823 (n = 64,450)	62	57	0.92 [0.28, 2.96]	8 [−196, 72]	0.88		0.92 [0.29, 2.98]
	All			129/156,982 (n = 129,973)	115/155,512 (n = 129,601)	82	74	0.91 [0.43, 1.91]		0.80		0.92 [0.44, 1.93]
Adults^c^	1	No	0	23/31,508 (n = 27,008)	20/30,269 (n = 26,222)	73	66	0.90 [0.15, 5.49]		0.91	0.98	0.91 [0.15, 5.56]
	2	Yes	0	11/27,243 (n = 23,238)	10/26,305 (n = 22,756)	40	38	1.00 [0.07, 14.79]		1.00		1.01 [0.07, 15.27]
	2^f^	Yes	0	25/35,972 (n = 56,329)	17/34,966 (n = 56,499)	69	49	0.70 [0.10, 4.88]		0.72		0.71 [0.10, 4.95]
	3	Yes	1	27/36,386 (n = 29,943)	14/37,213 (n = 30,329)	74	38	0.55 [0.08, 3.73]	45 [−273, 92]	0.54		0.55 [0.08, 3.78]
	4	Yes	2 and more	10/35,254 (n = 25,648)	12/35,353 (n = 26,067)	28	34	1.10 [0.08, 14.54]	−10 [−1354, 92]	0.94		1.11 [0.08, 15.09]
	1 & 2		0	34/58,751 (n = 50,246)	30/56,574 (n = 48,978)	58	53	0.92 [0.20, 4.16]		0.91		0.92 [0.20, 4.19]
	3 & 4	Yes	1 and more	37/71,640 (n = 55,591)	26/72,566 (n = 56,396)	52	36	0.71 [0.15, 3.25]	29 [−225, 85]	0.66		0.71 [0.15, 3.29]
	All			71/130,391 (n = 105,837)	56/129,140 (n = 105,374)	54	43	0.80 [0.27, 2.36]		0.69		0.81 [0.28, 2.38]
Children^c^	1	No	0	4/2217 (n = 2268)	1/2161 (n = 2229)	180	46	0.25 [0.00, 167.07]		0.68	0.16	0.24 [0.00, 159.96]
	2	Yes	0	41/15,563 (n = 14,247)	37/14,954 (n = 13,944)	263	247	0.93 [0.26, 3.28]		0.91		0.97 [0.27, 3.45]
	2^f^	Yes	0	48/16,538 (n = 18,081)	45/15,982 (n = 18,047)	290	282	1.00 [0.31, 3.24]		1.00		1.02 [0.31, 3.35]
	3	Yes	1	7/5337 (n = 4752)	12/5710 (n = 5018)	131	210	1.69 [0.12, 24.10]	−69 [−2310, 88]	0.70		1.59 [0.11, 22.98]
	4	Yes	2 and more	6/3474 (n = 2869)	9/3546 (n = 3036)	173	254	1.40 [0.07, 29.44]	−40 [−2844, 93]	0.83		1.67 [0.08, 34.93]
	1 & 2		0	45/17,780 (n = 16,515)	38/17,115 (n = 16,173)	253	222	0.82 [0.50, 1.37]		0.46		0.89 [0.26, 3.11]
	3 & 4	Yes	1 and more	13/8811 (n = 7621)	21/9256 (n = 8054)	148	227	1.46 [0.20, 10.86]	−46 [−986, 80]	0.71		1.50 [0.20, 11.08]
	All			58/26,591 (n = 24,136)	59/26,372 (n = 24,227)	218	224	1.00 [0.65, 1.54]		0.99		1.04 [0.36, 2.99]

a Vaccine coverage was defined as the number of TCV (or JE) vaccinees in every TCV (or JE) family.

b Blood-culture confirmed typhoid fever (no.)/Person-Years of follow up (number of residents).

c Adults was defined as residents aged over 18 years. Children were defined as residents aged 18 years or younger.

d Adjusted for stratification factors (the number of children 9 months to <16 years of age, ward and distance of cluster to the nearest health facility), follow-up period, covariates (age, gender, household toilet type, household source of drinking water, household type of drinking water, hand wash before meal, and hand wash after defecation), random effect (cluster, family), and family size.

e Interaction between number of vaccinees and arm for family groups 1,2,3,4 without group 2^f^.

f Including family groups 3 and 4 during the follow-up period between date of residence and the earliest vaccination date in the same family.

g Adjusted for stratification factors (the number of children 9 months to <16 years of age, ward and distance of cluster to the nearest health facility), follow-up period, covariates (age and gender), random effect (cluster, family), and family size.

**Table 3:** Incidence of blood culture-confirmed fever caused by other infection among non-vaccinees by family vaccine coverage.^a^

	Family group	With age-eligible children	Number of vaccinees	N/PYs (n)^b^		Incidence, per 100,000 PYs		Adjusted incidence rate ratio (%) [95% CI]^d^	P value	P value for interaction^e^
				JE	TCV	JE	TCV			
All family members	1	No	0	13/33,725 (n = 29,276)	5/32,431 (n = 28,451)	39	15	0.40 [0.14, 1.13]	0.09	0.57
	2	Yes	0	22/42,807 (n = 37,485)	14/41,259 (n = 36,700)	51	34	0.66 [0.34, 1.30]	0.23	
	3	Yes	1	14/41,723 (n = 34,695)	15/42,923 (n = 35,347)	34	35	1.02 [0.11, 9.74]	0.99	
	4	Yes	2 and more	7/38,728 (n = 28,517)	10/38,899 (n = 29,103)	18	28	1.58 [0.09, 28.51]	0.76	
Adults^c^	1	No	0	13/31,508 (n = 27,008)	5/30,269 (n = 26,222)	41	17	0.41 [0.02, 10.87]	0.60	0.70
	2	Yes	0	10/27,243 (n = 23,238)	8/26,305 (n = 22,756)	37	30	0.89 [0.05, 17.00]	0.94	
	3	Yes	1	12/36,386 (n = 29,943)	9/37,213 (n = 30,329)	33	24	0.77 [0.05, 12.10]	0.85	
	4	Yes	2 and more	6/35,253 (n = 25,648)	8/35,353 (n = 26,067)	17	23	1.37 [0.05, 39.46]	0.85	
Children^c^	1	No	0	0/2217 (n = 2268)	0/2161 (n = 2229)					0.61
	2	Yes	0	12/15,563 (n = 14,247)	6/14,954 (n = 13,944)	77	40	0.51 [0.02, 11.13]	0.67	
	3	Yes	1	2/5337 (n = 4752)	6/5710 (n = 5018)	37	105	2.81 [0.02, 379.63]	0.68	
	4	Yes	2 and more	1/3474 (n = 2869)	3/3546 (n = 3036)	29	85	3.03 [0.00, 3567.69]	0.76	

a Vaccine coverage was defined as the number of TCV (or JE) vaccinees in every TCV (or JE) family.

b Blood-culture confirmed fever by other infection (no.)/Person-Years of follow up (number of residents).

c Adults was defined as residents aged over 18 years. Children were defined as residents aged 18 years or younger.

d Adjusted for design variables (the number of children 9 months to <16 years of age, ward and distance of cluster to the nearest health facility), follow-up period, covariates (age, gender, household toilet type, household source of drinking water, household type of drinking water, hand wash before meal, and hand wash after defecation), random effect (family, cluster), and family size.

e Interaction between number of vaccinees and arm.

Among the vaccinated population, 91 typhoid fever episodes were detected among JE vaccinees, compared with 15 among TCV vaccinees in family group 3. The corresponding incidence rates were 574 and 93 per 100,000 person-years, respectively, yielding a TCV total vaccine effectiveness (VE) of 81% [95% CI: 21, 95] (Table 4). Similarly, in family group 4, typhoid incidence rates were 522 in the JE arm and 91 in the TCV arm, resulting in a VE of 79% [95% CI: 45, 92] (Table 4).

**Table 4:** Incidence of blood culture-confirmed typhoid among vaccinees by family vaccine coverage.^a^

	Family group	With age-eligible children	Number of vaccinees	N/PYs (n)^b^		Incidence, per 100,000 PYs		Adjusted incidence rate ratio (%) [95% CI]^c^	Vaccine effectiveness (%) [95% CI]^c^	P value	P value for interaction^d^
				JE	TCV	JE	TCV				
Vaccinees	3	Yes	1	91/15,852 (n = 11,443)	15/16,170 (n = 11,611)	574	93	0.19 [0.05, 0.79]	81 [21, 95]	0.02	0.90
	4	Yes	2 and more	171/32,760 (n = 21,955)	30/32,804 (n = 22,211)	522	91	0.21 [0.08, 0.55]	79 [45, 92]	<0.0001	

a Vaccine coverage was defined as the number of TCV (or JE) vaccinees in every TCV (or JE) family.

b Blood-culture confirmed typhoid (no.)/Person-Years of follow up (number of residents).

c Adjusted for design variables (the number of children 9 months to <16 years of age, ward and distance of cluster to the nearest health facility), follow-up period, covariates (age, gender, household toilet type, household source of drinking water, household type of drinking water, hand wash before meal, and hand wash after defecation), random effect (cluster, family), and family size.

d Interaction between number of vaccinees and arm.

A total of 29,505 children provided information about the schools they attended, among which 9209 attended the 35 schools with a size of >100 students in the study area. The TCV coverage rate across schools ranged from 8% to 55%, with a median coverage rate of 37%. Among non-TCV recipients, 22 typhoid fever episodes were detected in the low-coverage schools (≤median), compared with 14 in the high-coverage schools (>median) (Table 5). The incidence rates were 349 [95% CI: 219, 528] and 329 [95% CI: 180, 552] per 100,000 person-years in the low-coverage schools and high-coverage schools, respectively, with an aIRR of 0.96 [95% CI: 0.28, 3.37] (Table 5). For other infections, the incidence rates were 254 [95% CI: 145, 412] and 305 [95% CI: 163, 522] in the low-coverage schools and high-coverage schools, respectively, yielding an aIRR of 1.48 [95% CI: 0.65, 3.34] (Table 5). One and two typhoid fever episodes were reported among TCV recipients in low-coverage and high-coverage schools, respectively, with an aIRR of 2.13 [0.19, 23.46].

**Table 5:** Incidence of blood culture-confirmed typhoid fever and fever caused by other infection among children with school information by school TCV coverage.^a^

	N/PYs (n)^b^	Incidence, per 100,000 PYs [95% CI]	Adjusted incidence rate ratio [95% CI]^c^	P value	Adjusted incidence rate ratio (%) [95% CI] (simplified)^d^
Typhoid fever					
TCV recipients					
Below median	1/2450 (n = 1474)	41 [1, 227]	Ref		
Above median	2/2840 (n = 1673)	70 [9, 254]	2.13 [0.19, 23.46]	0.54	
Non-TCV recipients					
Below median	22/6305 (n = 4044)	349 [219, 528]	Ref		
Above median	14/4257 (n = 2812)	329 [180, 552]	0.96 [0.28, 3.37]	0.96	0.82 [0.30, 2.21]
Fever caused by other infection					
Non-TCV recipients					
Below median	16/6305 (n = 4044)	254 [145, 412]	Ref		
Above median	13/4257 (n = 2812)	305 [163, 522]	1.48 [0.65, 3.34]	0.35	1.58 [0.71, 3.53]

a Vaccine coverage was defined as the percentage of post-vaccination follow-up period of TCV vaccinees in follow-up period children with school information (including JE vaccinees). The median TCV coverage is 37%.

b Blood-culture confirmed fever (no.)/Person-Years of follow-up (number of children).

c Adjusted for geographical ward, distance to study clinics, number of eligible children at baseline, follow-up period, arm of school, age, sex, toilet type in the house, drinking water source, treatment of drinking water, and handwashing practices before meals and after defecation, and random effect of clusters and schools for non-TCV recipients; adjusted for follow-up period for TCV recipients due to limited number of cases.

d Adjusted for geographical ward, distance to study clinics, number of eligible children at baseline, follow-up period, arm of school, age, and sex, and random effect of clusters and schools for non-TCV recipients.

## Discussion

Our study did not show any statistically significant herd protection from immunisation with typhoid conjugate vaccine (TCV), regardless of vaccine coverage in households and schools. However, the estimates were imprecise, with wide confidence intervals, and should therefore be interpreted cautiously. While TCV's direct protection is well-established, the absence of indirect protection in our study highlights the challenge of achieving a significant herd effect in high-incidence, densely populated environments.

Several factors may contribute to the absence of herd protection in our study, primarily stemming from the strategy of vaccinating only children, particularly when contrasted with the earlier Vi polysaccharide vaccine cluster-randomised trial in Kolkata, where indirect protection was observed.^8^ A major difference between the two trials is overall population coverage, which resulted from different target populations. In Kolkata, residents aged 2 years and older were offered vaccination and approximately 61% of eligible individuals in the Vi vaccine clusters received the vaccine.^11^ In contrast, vaccine coverage in Bangladesh was 64% in children under 16, representing approximately 20% of residents. Given the high incidence of typhoid in Bangladesh (635 cases per 100,000 person-years in the control arm, observed in the original trial) this level of coverage may be insufficient to reach the 40–50% population-level threshold that modelling studies predict is needed to confer substantial indirect protection in South Asia and elsewhere.^6^^,^^12^ Taken together, these differences in target population and vaccine coverage are more likely to explain the absence of herd protection in our study than any intrinsic differences between the polysaccharide and conjugate vaccines.

Beyond the overall coverage, a child-only strategy may be inherently limited if adults are the primary drivers of transmission, for instance as food handlers or chronic carriers, even while children bear the highest disease burden. The inclusion of adults in the Kolkata trial may therefore have enhanced community-wide interruption of transmission and contributed to the observed indirect protection. These findings underscore that expanding vaccination to adults is essential for achieving effective population-level protection. An ongoing CRCT on the effectiveness of another TCV in India, which extended vaccination to individuals aged ≤30 years, may provide further insights on this matter between vaccine coverage and herd protection.

In addition to vaccine strategy, it is important to consider potential epidemiological factors that might explain our finding. One hypothesis is that the transmission in our trial setting may occur predominantly through shared environmental or food sources rather than direct person-to-person contact within family or school. This is a common characteristic of fecal-oral diseases. In such settings, vaccination of children alone may be insufficient to interrupt transmission, as environmental reservoirs can sustain the spread of the disease independently of human-to-human interactions. This hypothesis is supported by recent research in Bangladesh, where a novel method using bacteriophages as proxy trackers for *Salmonella* Typhi showed a strong correlation between environmental presence and blood culture-positive cases.^13^ Acknowledging this suggests that integrated approaches instead of relying solely on vaccination are needed to maximise its public health benefits. Combining vaccination with improvements in water, sanitation, and hygiene (WASH) infrastructure could address the environmental drivers of typhoid transmission and enhance the overall impact of vaccination programs. A secondary study of TyVAC Bangladesh showed that living in “Better WASH” households reduced typhoid risk by 37%, regardless of JE or TCV clusters [95% CI: 24%, 48%].^14^ In addition, although TCV is highly effective against symptomatic disease, its impact on asymptomatic infection and onward transmission has not been fully characterised, which can influence the indirect protection of TCV.

Several limitations to our study should be acknowledged. The primary limitation of this analysis is limited precision. The overall aIRR of 0.91 [95% CI: 0.43, 1.91] was compatible with a meaningful reduction in incidence as well as little or no indirect protection. Given the number of events observed among unvaccinated individuals, our study does not have enough power to detect modest levels of herd protection. Second, the distribution of vaccinated children across households was relatively homogeneous, with most families having 0, 1 or 2 vaccinated children due to the typical household size in the area, and few households reaching high coverage levels (e.g., 3+ vaccinated members). This clustering within a limited range of household vaccination status may have limited our ability to detect clear trends in herd protection when comparing groups. Third, data collection on school attendance was limited with only around 7000 children from large schools included in the school-level analysis, potentially reducing the power of school-level analyses. Besides, the analysis was restricted to schools with more than 100 students to ensure adequate cluster size and smaller schools were excluded. Vaccination coverage was not assessed in those schools. If smaller schools differed systematically in coverage or transmission patterns, this may limit the generalisability of the school-level findings. However, this limitation is unlikely to have introduced selection bias since incidence rates among TCV recipients and non-recipients in these 7000 children were consistent with incidence rates in all children from the original study as mentioned above.^6^ Besides, the school-level analysis relied on the full follow-up period, not just time spent at school, which includes significant time spent within the family where we found no herd protection. It means any school-specific effect was diluted, potentially limiting our power to detect a subtle protective effect in that environment. Additionally, while our study focused on households and schools, transient community interactions (e.g., markets, and public transit) were not captured in our design. It's important to recognise that transmission from parents, relatives, or caregivers in these environments may still be significant, even if children themselves have fewer interactions. Future studies of vaccination programs that include adults should consider these community spaces, as adult movement patterns may affect disease transmission beyond homes. Finally, our findings may not be generalisable to all settings, particularly remote areas where typhoid incidence rates are typically lower. In such areas, transmission dynamics may differ significantly from densely populated urban environments, but this remains a hypothesis that requires further investigation.

Future research on TCVs could explore integrating vaccination with environmental interventions, such as water quality monitoring and sanitation upgrades, to assess whether targeting both human and environmental transmission enhances herd protection. Comparative studies across diverse settings (e.g., urban slums vs. rural communities) could clarify how local WASH infrastructure modulates vaccine-derived herd effects. Geospatial mapping of high-risk transmission hubs (e.g., street-food markets, communal water points) could identify priority sites for coordinated vaccination and environmental measures, supported by cost-effectiveness analyses comparing integrated approaches and standalone interventions, including strategies focused solely on increasing vaccination coverage.

In conclusion, our study did not find significant herd protection in families and schools with different levels of vaccine coverage. In high-transmission urban settings similar to ours, expanding vaccination to the adult population may need further investigation to determine whether broader population coverage could interrupt ongoing transmission and enhance indirect effects. The complex interplay between vaccination, environmental factors, and local epidemiology highlights the need for multifaceted approaches to typhoid control and prevention. These findings help inform the development of more comprehensive strategies that integrate vaccination with targeted environmental interventions and enhanced surveillance efforts to effectively reduce the burden of typhoid fever globally.

## Contributors

FQ, JDC, AJP, and XL conceived the study design. YZ did the analyses with inputs from FA, FK, JDC, AJP, and XL. YZ and XL drafted this report. YFM and PKB designed and managed the study database. SK was the project manager. FQ, FK, and JDC led the data collection in the original trial for this report. All authors have read and approved the final version of this report and had final responsibility for the decision to submit for publication. All authors had full access to all the data in the study. YZ and XL directly accessed and verified the underlying data in this report.

## Data sharing statement

Deidentified individual participant data including a data dictionary for each variable analysed in this report will be made available when the original trial is complete, upon requests directed to the corresponding author. Only after approval of a proposal can data be shared through a secure online platform. Approval of the proposal will be subject to scientific review by the institutional review board at the International Centre for Diarrhoeal Disease Research, Bangladesh. Sharing of data will also be subject to the published data access rules of the International Centre for Diarrhoeal Disease Research, Bangladesh. The requestor will need to sign a standard data access agreement required by the International Centre for Diarrhoeal Disease Research, Bangladesh.

## Declaration of interests

AJP was the chair of the UK Department of Health and Social Care's Joint Committee on Vaccination until 2025; was a member of the WHOs Strategic Advisory Group of Experts until 2022; was the chair of WHOs TAG on Salmonella vaccines until end of 2024; and is a member of the WHO Product Development for Vaccines Advisory Committee from August 2025. AJP receives consulting fees from Shionogi and the Ellison Institute, Oxford. AJP, XL, and SK are contributors to intellectual property licenced by Oxford University Innovation to AstraZeneca. XL is a member of the WHO SAGE typhoid working group and a member of the WHO TAG on Salmonella vaccines. All other authors declare no competing interests. Oxford University has received funding for research on Salmonella vaccines from the Bill & Melinda Gates Foundation, the UK Medical Research Council, the Wellcome Trust, the European Commission, the Coalition for Epidemic Preparedness Innovations, the National Institute for Health and Care Research, AstraZeneca and the Serum Institute of India.
