# Supplementary material for: The association between vaccine coverage and herd protection: exploratory analyses of a cluster-randomised trial of Vi conjugate vaccine
Source: eClinicalMedicine. 2026 Apr 15;94:103863. doi: 10.1016/j.eclinm.2026.103863 (PMC13101550; doi:10.1016/j.eclinm.2026.103863)
Supplement: Supplementary Table [file mmc1.docx]

**Table S1 Incidence of blood culture-confirmed typhoid fever among non-vaccinees by family vaccine coverage***

|  | **Family**  **group** | **With age-eligible children** | **Proportion of vaccinees** | **N/PYs (n)^§^** | | **Incidence, per 100,000 PYs** | | **Adjusted**  **incidence rate ratio (%) [95%CI]^ꬷ^** | **Vaccine effectiveness** **(%) [95%CI]^ꬷ^** | **P value** |
| --- | --- | --- | --- | --- | --- | --- | --- | --- | --- | --- |
|  |  |  |  | **JE** | **TCV** | **JE** | **TCV** |  |  |  |
| All family members | 1 | No |  | 27/33,725 (n=29,276) | 21/32,431 (n=28,451) | 80 | 65 | 0.81 [0.15,4.52] |  | 0.81 |
|  | 2 | Yes | 0% | 52/42,807 (n=37,485) | 47/41,259 (n=36,700) | 121 | 114 | 0.97 [0.31,3.04] |  | 0.96 |
|  | 3 | Yes | 1%-50% | 14/13125 (n=10781) | 12/14100 (n=11366) | 107 | 85 | 0.79  [0.09,6.53] | 21 [-553,91] | 0.82 |
|  | 4 | Yes | 51%-100% | 36/67325 (n=52431) | 35/67723 (n=53084) | 53 | 52 | 0.96  [0.24,3.86] | 4 [-286,76] | 0.96 |
|  |  |  |  |  |  |  |  |  |  |  |
| Adults^#^ | 1 | No |  | 23/31,508 (n=27,008) | 20/30,269 (n=26,222) | 73 | 66 | 0.90 [0.15,5.49] |  | 0.91 |
|  | 2 | Yes | 0% | 11/27,243 (n=23,238) | 10/26,305 (n=22,756) | 40 | 38 | 1.00 [0.07,14.79] |  | 1.00 |
|  | 3 | Yes | 1%-50% | 7/9150 (n=7338) | 2/9780 (n=7650) | 77 | 20 | 0.24 [0.00,26.72] | 76  [-2572,100] | 0.55 |
|  | 4 | Yes | 51%-100% | 30/62490 (n=48253) | 24/62786 (n=48746) | 48 | 38 | 0.80 [0.16,4.12] | 20 [-312,84] | 0.79 |
|  |  |  |  |  |  |  |  |  |  |  |
| Unvaccinated children^#^ | 1 | No |  | 4/2,217  (n=2,268) | 1/2,161  (n=2,229) | 180 | 46 | 0.25 [0.00,167.07] |  | 0.68 |
|  | 2 | Yes | 0% | 41/15,563 (n=14,247) | 37/14,954 (n=13,944) | 263 | 247 | 0.93  [0.26,3.28] |  | 0.91 |
|  | 3 | Yes | 1%-50% | 7/3976 (n=3443) | 10/4320 (n=3716) | 176 | 232 | 1.21 [0.08,18.69] | -21  [-1769,92] | 0.89 |
|  | 4 | Yes | 51%-100% | 6/4835 (n=4178) | 11/4937 (n=4338) | 124 | 223 | 1.73 [0.09,33.02] | -73  [-3202,91] | 0.72 |

* Vaccine coverage was defined as the proportion of TCV (or JE) vaccinees among age-eligible children in every TCV (or JE) family

**^§^** Blood-culture confirmed typhoid fever (no.)/ Person-Years of follow up (number of residents)

^#^ Adults was defined as residents aged over 18 years. Children were defined as residents aged 18 years or younger.

**^ꬷ^** Adjusted for stratification factors (the number of children 9 months to <16 years of age, ward and distance of cluster to the nearest health facility), follow-up period, covariates (age, gender, household toilet type, household source of drinking water, household type of drinking water, hand wash before meal, and hand wash after defecation), random effect (cluster, family), and family size
